# The distribution of pelvic organ support defects in women undergoing pelvic organ prolapse surgery and compartment specific risk factors

**DOI:** 10.1007/s00192-021-04826-7

**Published:** 2021-05-11

**Authors:** Emmanuel Payebto Zoua, Michel Boulvain, Patrick Dällenbach

**Affiliations:** 1 Department of Pediatrics, Gynecology and Obstetrics, Division of Gynecology, Urogynecology Unit, Geneva University Hospitals, 30 boulevard de la Cluse, 1211 Genève 14, Switzerland; 2 Faculty of Medicine, University of Geneva, Geneva, Switzerland

**Keywords:** Observational study, Pelvic organ prolapse, Distribution of pelvic organ prolapse, Pelvic support defect, Pelvic organ prolapse surgery

## Abstract

**Introduction and hypothesis:**

The objective of our study was to describe the distribution of pelvic organ prolapse (POP) in a population of women undergoing POP reconstructive surgery and to identify compartment-specific risk factors.

**Methods:**

We conducted a retrospective observational study in a cohort of 326 women who underwent POP repair and had a standardized preoperative POP assessment using the Baden-Walker classification. The distribution of POP grade was described for each vaginal compartment. The association between the involvement of each specific compartment and predictors was evaluated with a logistic regression model.

**Results:**

The frequency of significant POP (grade ≥ 2) was 79% in the anterior compartment, 49% in the middle/apical compartment and 31% in the posterior compartment. Combined significant anterior and apical defects were present in 25% of women. Increasing age was a significant risk factor for apical defect (between 60 and 70 years OR = 2.4, 95% CI 1.2–4.6; > 70 years OR = 3.4, 95% CI 1.7–6.6). Previous hysterectomy (OR = 2.2, 95% CI 1.0–4.6) was a significant risk factor for posterior defect.

**Conclusions:**

In a population undergoing POP surgery, anterior compartment involvement is the most common and serious defect and can often be associated with an apical defect, especially in older women. In case of previous hysterectomy, the posterior compartment may be weakened. These findings may help surgeons to select the appropriate POP reconstructive surgery, which often should address both anterior and apical defects.

**Supplementary Information:**

The online version contains supplementary material available at 10.1007/s00192-021-04826-7.

## Introduction

Pelvic organ prolapse (POP) is a frequent condition affecting up to 50% of women, and the prevalence increases with age [1]. The lifetime risk of undergoing POP surgery is estimated to be between 6.3% and 19% [2–4]. There are numerous pelvic floor reconstructive surgical techniques. The risk of reoperation for recurrence is estimated to be between 10 and 30% [2–4]. To limit the risk of recurrence, understanding of pelvic floor defect distribution and the potential risk factors is important in choosing the best surgical procedure wisely. The distribution of POP defects was described in a few studies in the general population, but to our knowledge only once in a cohort of women undergoing POP reconstructive surgery [2, 5, 6].

The purpose of this study was to describe the distribution of pelvic floor defects among the anterior, apical and posterior compartments in a population of women undergoing POP reconstructive surgery and to identify specific risk factors for each one of them.

## Subjects and methods

We performed an observational retrospective cohort study including 326 women undergoing reconstructive POP surgery. We used data from a previous study approved by the Institutional Ethics Committee of the Geneva University Hospitals (protocol number 07-242R). This sample was described in a previous report [7]. Briefly, the sample was randomly selected to be representative of a larger cohort of 1811 women who underwent POP repair in our department between 1988 and 2007. Demographic and medical data of all women were retrieved from their medical charts and were stored anonymously in a computer database. Variables included age, weight and height, parity, number of vaginal deliveries, menopausal status, the presence of diabetes, heart disease, smoking, asthma, chronic obstructive pulmonary disease (COPD), constipation and sexual activity, and history of hysterectomy or previous surgery for POP or urinary incontinence. All women had a standardized preoperative POP assessment, using the Baden-Walker classification, which was the classification system used in our institution during the study period [8]. The presence and the grade of anterior, apical and posterior prolapse was available for all 326 women.

We analyzed the distribution of pelvic floor defects in each vaginal compartment and identified specific risk factors for each one of them. We described the distribution of the global POP grade (defined as the most severe grade across all compartments) and the distribution of the POP grade in each compartment separately. We used the Friedman test to compare the distribution of POP grade in the three compartments.

We analyzed the prevalence of clinically significant pelvic support defects (defined as POP grade ≥ 2) in specific compartments among women undergoing surgery. We also analyzed the proportion of defects involving a single, two or all compartments. For the three specific compartments, we tested the association between the presence of POP grade ≥ 2 and potential predictors. Potential predictors included: age (< 50, 50 to 59, 60 to 69, ≥ 70), BMI (categories according to the quantiles of the distribution: < 23.5, 23.5 to 25.4, 25.5 to 29, ≥ 29), parity (< 3 and ≥ 3), history of vaginal delivery, history of surgery for POP or UI, history of hysterectomy and presence of COPD. We performed a univariate analysis to compute compartment-specific odds ratios (OR) with their 95% confidence intervals (CI). To evaluate the independent contribution of the identified predictors, we used a multivariable logistic regression. A *p* value < 0.05 was considered to indicate statistical significance.

We used SPSS 23.0 statistical software (SPSS Inc., Chicago, IL, USA) for data handling and statistical analysis.

## Results

The characteristics of the 326 women undergoing POP reconstructive surgery are shown in Table 1. Mean age was 61 years (SD 13) ranging from 31 to 91 years. Mean BMI was 26.4 kg/m^2^ (SD 4.4), 237 women (72%) were postmenopausal, 43 (13%) had a history of hysterectomy, and 35 (10.3%) already had undergone previous surgery for POP or urinary incontinence (UI) at the time of surgery. For 13 women, BMI was missing, for 40 women sexual activity and constipation were missing, and other characteristics were occasionally missing.

**Table 1:** Characteristics of the study population

Characteristics	*N* = 326
Age (years) mean (SD)	61.3 (13)
BMI (kg/m^2^) mean (SD)^a^	26.4 (4.4)
Overweight (BMI > 25) *n* (%)	168 (51.5)
Obese (BMI > 30) *n* (%)	57 (17.5)
Parity mean (SD)^b^	
0 *n* (%) 1 *n* (%) 2 *n* (%) ≥ 3 *n* (%)	3.2 (1.1) 13 (4.0) 68 (20.9) 140 (43.1) 103 (31.7)
Previous cesarean *n* (%)	10 (3.1)
Previous surgery for POP or UI *n* (%)	35 (10.7)
History of hysterectomy *n* (%)^c^	43 (13.2)
Smoking > 5 cigarettes/day, *n* (%)^d^	40 (12.4)
COPD *n* (%)^b^	6 (1.8)
Diabetes *n* (%)^b^	26 (8.0)
Cardiovascular disease *n* (%)^c^	21 (6.5)
Constipation *n* (%)^e^	90 (31.5)
Sexual activity *n* (%)^e^	173 (60.5)

Data are presented as mean (SD) and *n* (%)

*SD* standard deviation, *BMI* body mass index, *COPD* chronic obstructive pulmonary disease

^a,b,c,d,e^There were respectively 13, 1, 2, 3 and 40 missing values for these characteristics

**Fig. 1:**
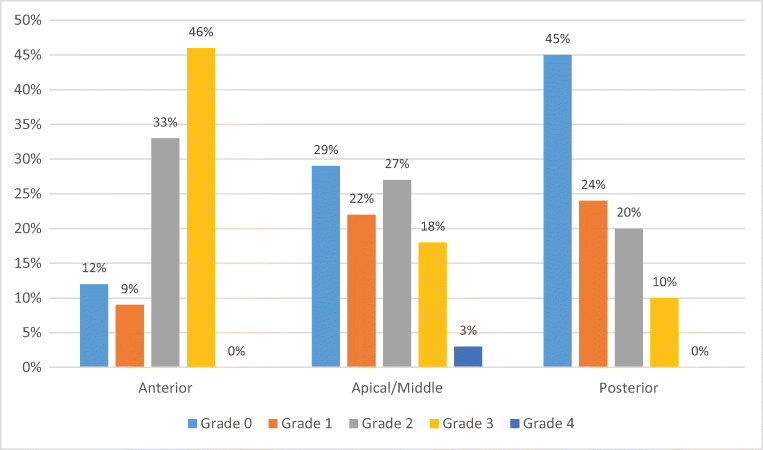
Grade distribution of POP in the three vaginal compartments among women undergoing POP surgery

The global POP grade distribution was 2.2% of POP grade 1, 32.5% grade 2, 62.0% grade 3 and 3.7% grade 4. The POP grade distribution for the three separate compartments is shown in Fig. 1. There was some defect (≥ grade 1) in the anterior compartment in 88%, in the apical/middle compartment prolapse in 71% and in the posterior compartment in 55% of women. The anterior compartment was the most severely affected with most women (46%) having a POP of grade 3 in this compartment. In the middle/apical compartment the distribution was uniform among grades 1, 2 and 3. The posterior compartment was the least severely affected with most women having no defect (45%) or POP grade 1 (24%). A POP of grade 4 was present in the middle compartment only. The grade distributions in the three compartments were significantly different (Friedman’s test *P* < 0.001).

Using POP grade ≥ 2 as a threshold for defining clinically significant defects, the proportion of women with anterior, middle and posterior compartment defects was 78.8%, 49.1% and 31.3%, respectively. Analyzing significant multiple compartment defects with the same threshold, we found that 50% of women had a defect in only one compartment, 35% in two and 13% in all three compartments (Table 2). The most frequent combined defect involved the anterior and middle compartments. Seven women of our cohort (2%) were operated for grade 1 POP.

**Table 2:** Distribution of POP grade ≥ 2 for single or multiple compartment defects

Compartment(s)	*N* = 326
Grade 1	7 (2.1%)
Anterior only	108 (33.1%)
Middle only	28 (8.6%)
Posterior only	26 (8%)
Anterior and middle	81 (24.8%)
Anterior and posterior	25 (7.7%)
Middle and posterior	8 (2.5%)
All three compartments	43 (13.2%)
Total	326 (100%)

Data are presented as n (%)

The univariable analysis of predictors for an anterior compartment defect showed history of operation for POP or UI (OR 0.3, 95% CI 0.2–0.7) as decreasing the risk. For the middle/apical compartment there was an association between increasing age (relative to women younger than 50 years) and the presence of a defect: age 60 to 69 (OR 2.1, 95% CI 1.1–3.9); age ≥ 70 (OR 3.0, 95% CI 1.6–5.6) (Table S2). History of operation for POP or UI (OR 2.9, 95% CI 1.4–6.1) and history of hysterectomy (OR 2.6, 95% CI 1.4–5.0) increased the risk of defect in the posterior compartment. BMI ≥ 29 (OR 1.73; 95% CI 0.9–3.4) (relative to women with BMI < 23.5) and history of vaginal delivery (OR 1.9, 95% CI 0.5–6.9) also increased the risk of posterior defect, but their effects were not statistically significant. In the multivariable analysis, we found a statistically significant association with the presence of clinically significant POP for the following variables. Previous surgery for POP or UI remained protective for an anterior compartment defect (adjusted OR 0.4; 95% CI 0.2–0.90). Age 60 to 69 (adjusted OR 2.4; 95% CI 1.2–4.6) and age ≥ 70 (adjusted OR 3.4; 95% CI 1.7–6.6) remained risk factors for POP development in the apical/middle compartment, relative to women younger than 50 years. Personal history of hysterectomy (adjusted OR 2.2; 95% CI 1.0–4.6) remained a significant risk factor for POP development in the posterior compartment. History of surgery for POP/UI was no longer a statistically significant predictor of posterior defect (OR 1.9, 95% CI 0.8–4.4), because many women had both history of POP/UI surgery and hysterectomy (18 women among the 58 who underwent surgery).

## Discussion

Our study suggests that in a population of women undergoing surgery for symptomatic POP, the anterior compartment is the most frequently and severely involved and may often be associated with a middle/apical defect. Increasing age was a significant risk factor for apical defect, and previous hysterectomy significantly increased the risk for posterior defect.

To our knowledge, only one study has described the distribution of pelvic floor defects according to location and severity in a population of 384 women undergoing surgery for a pelvic organ prolapse or for urinary incontinence. In this retrospective cohort study from Olsen et al., the population characteristics were similar to ours [2]. It is difficult to compare our data with theirs, as there were many missing values for preoperative POP assessment in their study. However, in Olsen’s study, 82%, 37% and 46% of the women had a repair in the anterior, middle and posterior compartment, respectively, which is similar to the frequency of POP of grade ≥ 2 in our study. Many authors agree that clinically significant (and symptomatic) POPs are the ones reaching or overpassing the hymen, which correspond to POP grade ≥ 2 [9]. A limitation in Olsen’s study was that the method of assessing preoperative POP was not standardized. Their data included descriptive terms used by the operating gynecologist and urologist to assess the POP grade in each compartment. Previous hysterectomy, which is a risk factor for posterior compartment prolapse in our study, was also not available in Olsen’s report. Yet, we note that they too found anterior defects to be the most frequent.

In a prospective cohort study, Ellerkmann et al. investigated the distribution of pelvic floor defects in a population of women visiting a clinic with complaints of symptomatic pelvic floor defects [10]. This study also found the anterior compartment to be the most frequently affected with 68% of women having POP ≥ grade 2. Surprisingly, and in agreement with Olsen et al., it also found posterior POP to be more common (prevalence estimated to be 60%) than apical ones (prevalence estimated to be 26%), which is in contradiction with our results [2]. However, 46% of the women in their study had a history of hysterectomy compared to only 13% in ours. Since we found that previous hysterectomy is a risk factor for the development of POP in the posterior compartment, this could explain this difference.

Our study also showed that significant defects involved two compartments or more in almost half of the women, with defects involving both the anterior and apical compartment being the most frequent. Based on these observations, we believe that the surgical technique should most often address the anterior and apical compartment together. Laparoscopic repair of POP by lateral suspension with mesh and uterine conservation may be a valuable technique for POP repair regarding these observations [11, 12]. As a posterior compartment defect is less common and is best treated by a vaginal approach without mesh, we believe it can be treated during the same operation, if required, or secondarily with a standard posterior colporrhaphy if it appears later [13–15]. In addition, uterine preservation in itself may prevent the further development of posterior compartment prolapse.

This study identified compartment specific determinants for the presence of a significant prolapse. For the apical compartment, we found that increasing age is a risk factor for the presence of significant prolapse. Previous studies also found age to be associated with an increased risk of POP; however, this study indicates that this is specifically associated with POP development in the apical compartment [5, 16–18]. Organ support for the cervix and the upper third of the vagina is provided mainly by the cardinal and the uterosacral ligaments. DeLancey suggested that the failure of these structures contributes to the formation of apical prolapse and that their length is a strong predictor for the presence of a defect [19]. Ramanah et al. in their review of anatomy and histology of apical support showed that cardinal and uterosacral ligaments are in fact mainly visceral structures consisting of vessels and nerves, but also connective and adipose tissues [20]. A possible hypothesis is that, in susceptible women, these structures may elongate with age because of a decrease in smooth muscle fibers and connective tissue [21].

Another important finding of this study is that a history of hysterectomy increases the risk of POP in the posterior compartment. Our observation is supported by that of Lykke et al., who analyzed POP repair subsequent to hysterectomy in 5279 women with radical hysterectomy and 149,920 women with total abdominal hysterectomy. They found the distribution of POP operations in relation to compartments to be predominant for the posterior one (50%) compared to the anterior (40%) and the apical compartments (10%) [22]. This is coherent with the anatomy of pelvic support described by Perucchini et al. [23]. When performing this surgery, the cardinal ligaments and paracolpium are sectioned. The vaginal vault is sutured and solidarized to the uterosacral ligament remnants. Despite the suture, the pelvic floor support is altered, in particular the connection between the paracolpium and the upper one-third of the posterior vagina, which may increase the risk of posterior compartment prolapse, as described by DeLancey [23]. Luo et al., using a computer model, also suggested that altering the apical support by reducing the uterosacral and cardinal ligament stiffness, together with an alteration of the levator ani function, led to POP involving the posterior compartment [24].

The limitations of our study are those of studies based on information collected in medical records, with potential missing data. In the history of women with previous POP or UI repair, we had no information regarding which compartment was involved during previous surgery, which could have been of interest.

The strength of our study was the availability of a detailed computerized register with preoperative standardized POP assessment according to the Baden-Walker classification.

In conclusion, our study shows that in a population of women undergoing POP surgery, anterior and apical defects are the most frequent and severe. These defects often occur together, and therefore the surgical technique should be able to repair both during the same procedure. Apart from women with prior hysterectomy, posterior pelvic floor defects are less frequent, and often less severe, and may be treated by standard posterior colporrhaphy.

## Supplementary Information

ESM 1 (DOCX 26 kb)

## Abbreviations

POP: Pelvic organ prolapse
OR: Odds ratio
CI: Confidence interval
BMI: Body mass index
HT: Hormonal replacement therapy
UI: Urinary incontinence
TH: Total hysterectomy
SH: Subtotal hysterectomy
VH: Vaginal hysterectomy
AH: Abdominal hysterectomy
LAVH: Laparoscopically assisted VH
ASCP: Abdominal sacrocolpopexy
LSCP: Laparoscopic sacrocolpopexy

## References

[1] Barber MD, Maher C (2013). Epidemiology and outcome assessment of pelvic organ prolapse. Int Urogynecol J.

[2] Olsen A, Smith V, Bergstrom J (1997). Epidemiology of surgically managed pelvic organ prolapse and urinary incontinence. Obstet Gynecol.

[3] Fialkow MF, Newton KM, Lentz GM, Weiss NS (2008). Lifetime risk of surgical management for pelvic organ prolapse or urinary incontinence. Int Urogynecol J Pelvic Floor Dysfunct.

[4] Smith FJ, Holman CDJ, Moorin RE, Tsokos N (2010). Lifetime risk of undergoing surgery for pelvic organ prolapse. Obstet Gynecol.

[5] Swift S, Woodman P, O’Boyle A (2005). Pelvic Organ Support Study (POSST): the distribution, clinical definition, and epidemiologic condition of pelvic organ support defects. Am J Obstet Gynecol.

[6] Hendrix SL, Clark A, Nygaard I (2002). Pelvic organ prolapse in the Women’s Health Initiative: gravity and gravidity. Am J Obstet Gynecol.

[7] Dällenbach P, Jungo Nancoz C, Eperon I (2012). Incidence and risk factors for reoperation of surgically treated pelvic organ prolapse. Int Urogynecol J.

[8] Baden WF, Walker T (1992). Surgical repair of vaginal defects.

[9] Swift SE, Tate SB, Nicholas J (2003). Correlation of symptoms with degree of pelvic organ support in a general population of women: what is pelvic organ prolapse?. Am J Obstet Gynecol.

[10] Ellerkmann RM, Cundiff GW, Melick CF (2001). Correlation of symptoms with location and severity of pelvic organ prolapse. Am J Obstet Gynecol.

[11] Dällenbach P, Veit N (2014). Robotically assisted laparoscopic repair of anterior vaginal wall and uterine prolapse by lateral suspension with mesh: initial experience and video. Int Urogynecol J.

[12] Martinello R, Scutiero G, Stuto A (2019). Correction of pelvic organ prolapse by laparoscopic lateral suspension with mesh: a clinical series. Eur J Obstet Gynecol Reprod Biol.

[13] Mowat A, Maher D, Baessler K (2018). Surgery for women with posterior compartment prolapse. Cochrane Database Syst Rev.

[14] Paraiso MFR, Barber MD, Muir TW, Walters MD (2006). Rectocele repair: a randomized trial of three surgical techniques including graft augmentation. Am J Obstet Gynecol.

[15] Kuhn A, Gelman W, O’Sullivan S, Monga A (2006). The feasibility, efficacy and functional outcome of local anaesthetic repair of anterior and posterior vaginal wall prolapse. Eur J Obstet Gynecol Reprod Biol.

[16] Progetto Menopausa Italia Study Group (2000). Risk factors for genital prolapse in non-hysterectomized women around menopause. Results from a large cross-sectional study in menopausal clinics in Italy. Eur J Obstet Gynecol Reprod Biol.

[17] Nygaard I, Bradley C, Brandt D, Women’s Health Initiative (2004). Pelvic organ prolapse in older women: prevalence and risk factors. Obstet Gynecol.

[18] Whitcomb EL, Rortveit G, Brown JS (2009). Racial differences in pelvic organ prolapse. Obstet Gynecol.

[19] DeLancey JOL (2016). What’s new in the functional anatomy of pelvic organ prolapse?. Curr Opin Obstet Gynecol.

[20] Ramanah R, Berger MB, Parratte BM, DeLancey JOL (2012). Anatomy and histology of apical support: a literature review concerning cardinal and uterosacral ligaments. Int Urogynecol J.

[21] Chen G-D (2007). Pelvic floor dysfunction in aging women. Taiwan J Obstet Gynecol.

[22] Lykke R, Blaakær J, Ottesen B, Gimbel H (2017). Incidence of pelvic organ prolapse repair subsequent to hysterectomy: a comparison between radical hysterectomy and total abdominal hysterectomy. Int Urogynecol J.

[23] Perucchini D, DeLancey JOL, Baessler K, Burgio KL, Norton PA (2008). Functional anatomy of the pelvic floor and lower urinary tract. Pelvic floor re-education.

[24] Luo J, Chen L, Fenner DE (2015). A multi-compartment 3-D finite element model of rectocele and its interaction with cystocele. J Biomech.

