# Supplementary material for: The distribution of pelvic organ support defects in women undergoing pelvic organ prolapse surgery and compartment specific risk factors
Source: Int Urogynecol J. 2021 May 11;33(2):405–9. doi: 10.1007/s00192-021-04826-7 (PMC8803792; doi:10.1007/s00192-021-04826-7)
Supplement: Supplementary file 1 — (DOCX 26 kb) [file 192_2021_4826_MOESM1_ESM.docx]

**Supplementary material**

**Table S1**: Univariable and multivariable analysis for the presence of POP grade ≥ 2 in the anterior compartment (n = 323) ^a^.

|  | n (%) | Crude OR (95 % CI) | P | Adjusted OR (95 CI %) | P |
| --- | --- | --- | --- | --- | --- |
| Age |  |  |  |  |  |
| < 50 (n = 75) | 58 (77.3) |  |  |  |  |
| 50 ≤ age < 60 (n = 72) | 60 (82.2) | 1.35 (0.60 – 3.03) | 0.463 | 1.16 (0.49 - 2.78) |  |
| 60 ≤ age < 70 (n = 84) | 69 (82.1) | 1.35 (0.62 – 2.93) | 0.451 | 1.29 (0.56 -2.96) | 0.549 |
| ≥ 70 (n = 91) | 68 (74.7) | 0.87 (0.42 – 1.77) | 0.696 | 0.71 (0.33 – 1.56) | 0.394 |
| BMI |  |  |  |  |  |
| < 23.5 (n = 82) | 65 (79.3) |  |  |  |  |
| 23.5 ≤ BMI < 25.5 (n = 73) | 58 (79.5) | 1.01 (0.46 – 2.21) | 0.977 | 1.03 (0.47 – 2.28) | 0.942 |
| 25.5 ≤ BMI < 29 (n = 74) | 67 (90.5) | 2.50 (0.97 – 6.43) | 0.057 | 2.35 (0.90 – 6.13) | 0.082 |
| ≥ 29 (n = 80) | 57 (71.3) | 0.65 (0.32 – 1.33) | 0.238 | 0.62 (0.29 – 1.32) | 0.212 |
| Unknown (n=14) | 8 (57.1) | 0.35 (0.11 – 1.14) | 0.082 | 0.35 (0.10 – 1.23) | 0.101 |
| Parity ^b^ |  |  |  |  |  |
| ≤ 2 (n = 220) | 171 (77.7) |  |  |  |  |
| > 2 (n =103) | 84 (81.6) | 1.27 (0.70 – 2.29) | 0.432 | 1.25 (0.66 – 2.35) | 0.498 |
| Vaginal delivery ^b^ |  |  |  |  |  |
| No history of (n = 15) | 12 (80.0) |  |  |  |  |
| History of (n = 308) | 243 (78.9) | 0.94 (0.26 – 3.41) | 0.918 | 1.06 (0.27 – 4.20) | 0.931 |
| Previous surgery for POP or UI |  |  |  |  |  |
| No history of (n = 290) | 236 (81.4) |  |  |  |  |
| History of (n=33) | 19 (57.6) | 0.31 (0.15 – 0.66) | 0.002 | 0.36 (0.15 – 0.90) | 0.025 |
| Hysterectomy ^b^ |  |  |  |  |  |
| No history of (n=280) | 224 (80.0) |  |  |  |  |
| History of (n= 43) | 31 (72.1) | 0.65 (0.31 – 1.34) | 0.239 | 1.01 (0.41 – 2.51) | 0.981 |
| COPD ^b^ |  |  |  |  |  |
| Absence of (n=317) | 250 (78.9) |  |  |  |  |
| Presence of (n=6) | 5 (83.3) | 1.34 (0.15 – 11.67) | 0.791 | 2.11 (2.23 – 19.44) | 0.512 |

**Table S2:** Univariable and multivariable analysis for the presence of POP grade ≥ 2 in the apical/middle compartment (n = 323) ^a^.

|  | n (%) | Crude OR (95 % CI) | P | Adjusted OR (95 CI %) | P |
| --- | --- | --- | --- | --- | --- |
| Age |  |  |  |  |  |
| < 50 (n = 75) | 27 (36.0) |  |  |  |  |
| 50 ≤ age < 60 (n = 72) | 28 (38.4) | 1.11 (0.57 – 2.16) | 0.767 | 1.22 (0.61 – 2.45) | 0.569 |
| 60 ≤ age < 70 (n = 84) | 45 (53.6) | 2.05 (1.09 – 3.89) | 0.027 | 2.37 (1.22 – 4.58) | 0.011 |
| ≥ 70 (n = 91) | 57 (62.6) | 2.98 (1.58 – 5.62) | 0.001 | 3.38 (1.74 – 6.59) | 0.000 |
| BMI |  |  |  |  |  |
| < 23.5 (n = 82) | 43 (52.4) |  |  |  |  |
| 23.5 ≤ BMI < 25.5 (n = 73) | 34 (46.6) | 0.79 (0.42 – 1.49) | 0.466 | 0.70 (0.36 – 1.35) | 0.288 |
| 25.5 ≤ BMI < 29 (n = 74) | 37 (50.0) | 0.91 (0.48 – 1.70) | 0.761 | 0.88 (0.45 -1.70) | 0.698 |
| ≥ 29 (n = 80) | 36 (45.0) | 0.74 (0.40 – 1.38) | 0.344 | 0.86 (0.45 – 1.65) | 0.647 |
| Unknown (n=14) | 7 (50.0) | 0.91 (0.29 – 2.81) | 0.866 | 1.12 (0.34 – 3.74) | 0.850 |
| Parity ^b^ |  |  |  |  |  |
| ≤ 2 (n = 220) | 106 (48.2) |  |  |  |  |
| > 2 (n =103) | 51 (45.5) | 1.06 (0.66 – 1.68) | 0.823 | 1.04 (0.63 – 1.71) | 0.889 |
| Vaginal delivery ^b^ |  |  |  |  |  |
| No history of (n = 15) | 9 (60.0) |  |  |  |  |
| History of (n = 308) | 148 (48.1) | 0.63 (0.21 – 1.77) | 0.370 | 0.71 (0.23 – 2.20) | 0.548 |
| Previous surgery for POP or UI |  |  |  |  |  |
| No history of (n = 290) | 142 (49.9) |  |  |  |  |
| History of (n=33) | 15 (45.5) | 0.87 (0.42 – 1.79) | 0.702 | 1.05 (0.46 – 2.43) | 0.909 |
| Hysterectomy ^b^ |  |  |  |  |  |
| No history of (n=280) | 139 (49.6) |  |  |  |  |
| History of (n= 43) | 18 (41.9) | 0.73 (0.38 – 1.40) | 0.343 | 0.57 (0.27 – 1.21) | 0.141 |
| COPD ^b^ |  |  |  |  |  |
| Absence of (n=317) | 155 (48.9) |  |  |  |  |
| Presence of (n=6) | 2 (33.3) | 0.52 (0.09 – 2.89) | 0.457 | 0.36 (0.06 – 2.15) | 0.265 |

**Table S3**: Univariable and multivariable analysis for the presence of POP grade ≥ 2 in the posterior compartment (n = 323) ^a^.

|  | n (%) | Crude OR (95 % CI) | P | Adjusted OR (95 CI %) | P |
| --- | --- | --- | --- | --- | --- |
| Age |  |  |  |  |  |
| < 50 (n = 75) | 23 (30.7) |  |  | 0.90 (0.43 – 1.89) | 0.776 |
| 50 ≤ age < 60 (n = 72) | 22 (30.1) | 0.98 (0.48 – 1.97) | 0.944 | 0.82 (0.40 – 1.68) | 0.583 |
| 60 ≤ age < 70 (n = 84) | 25 (29.8) | 0.96 (0.49 – 1.89) | 0.901 | 1.21 (0.61 – 2.43) | 0.585 |
| ≥ 70 (n = 91) | 32 (35.2) | 1.23 (0.64 – 2.36) | 0.540 |  |  |
| BMI |  |  |  |  |  |
| < 23.5 (n = 82) | 22 (26.8) |  |  |  |  |
| 23.5 ≤ BMI < 25.5 (n = 73) | 23 (31.5) | 1.26 (0.63 – 2.51) | 0.522 | 1.24 (0.61 – 2.53) | 0.554 |
| 25.5 ≤ BMI < 29 (n = 74) | 20 (27.0) | 1.01 (0.50 – 2.05) | 0.978 | 0.99 (0.48 – 2.05) | 0.981 |
| ≥ 29 (n = 80) | 31 (38.8) | 1.73 (0.89 – 3.35) | 0.107 | 1.63 (0.81 – 3.25) | 0.170 |
| Unknown (n=14) | 6 (42.9) | 2.05 (0.64 – 6.56) | 0.229 | 1.75 (0.51 – 5.94) | 0.371 |
| Parity ^b^ |  |  |  |  |  |
| ≤ 2 (n = 220) | 70 (31.8) |  |  |  |  |
| > 2 (n =103) | 32 (31.1) | 0.97 (0.58 – 1.60) | 0.892 | 0.93 (0.53 – 1.58) | 0.778 |
| Vaginal delivery ^b^ |  |  |  |  |  |
| No history of (n = 15) | 3 (20.0) |  |  |  |  |
| History of (n = 308) | 99 (32.1) | 1.90 (0.52 – 6.87) | 0.331 | 2.00 (0.52 – 7.77) | 0.314 |
| Previous surgery for POP or UI |  |  |  |  |  |
| No history of (n = 290) | 84 (29.0) |  |  |  |  |
| History of (n=33) | 18 (54.5) | 2.94 (1.42 – 6.11) | 0.004 | 1.89 (0.82 – 4.35) | 0.132 |
| Hysterectomy ^b^ |  |  |  |  |  |
| No history of (n=280) | 80 (26.6) |  |  |  |  |
| History of (n= 43) | 22 (51.2) | 2.62 (1.37 – 5.03) | 0.004 | 2.16 (1.01 – 4.59) | 0.046 |
| COPD ^b^ |  |  |  |  |  |
| Absence of (n=317) | 100 (31.5) |  |  |  |  |
| Presence of (n=6) | 2 (33.3) | 1.09 (1.20 – 6.02) | 0.926 | 0.81 (0.140 – 4.744) | 0.819 |

^a^ There were 3 missing values, which were excluded from both the univariable and the multivariable analysis. ^b^ A value was missing for these variable.
